# Rapid Removal of Tetrabromobisphenol A by Ozonation in Water: Oxidation Products, Reaction Pathways and Toxicity Assessment

**DOI:** 10.1371/journal.pone.0139580

**Published:** 2015-10-02

**Authors:** Ruijuan Qu, Mingbao Feng, Xinghao Wang, Qingguo Huang, Junhe Lu, Liansheng Wang, Zunyao Wang

**Affiliations:** 1 State Key Laboratory of Pollution Control and Resources Reuse, School of the Environment, Nanjing University, Jiangsu Nanjing, P. R. China; 2 College of Agricultural and Environmental Sciences, Department of Crop and Soil Sciences, University of Georgia, Griffin, Georgia, United States of America; 3 College of Resources and Environmental Science, Nanjing Agriculture University, Nanjing, P. R. China; Chinese Research Academy of Environmental Sciences, CHINA

## Abstract

Tetrabromobisphenol A (TBBPA) is one of the most widely used brominated flame retardants and has attracted more and more attention. In this work, the parent TBBPA with an initial concentration of 100 mg/L was completely removed after 6 min of ozonation at pH 8.0, and alkaline conditions favored a more rapid removal than acidic and neutral conditions. The presence of typical anions and humic acid did not significantly affect the degradation of TBBPA. The quenching test using isopropanol indicated that direct ozone oxidation played a dominant role during this process. Seventeen reaction intermediates and products were identified using an electrospray time-of-flight mass spectrometer. Notably, the generation of 2,4,6-tribromophenol was first observed in the degradation process of TBBPA. The evolution of reaction products showed that ozonation is an efficient treatment for removal of both TBBPA and intermediates. Sequential transformation of organic bromine to bromide and bromate was confirmed by ion chromatography analysis. Two primary reaction pathways that involve cleavage of central carbon atom and benzene ring cleavage concomitant with debromination were thus proposed and further justified by calculations of frontier electron densities. Furthermore, the total organic carbon data suggested a low mineralization rate, even after the complete removal of TBBPA. Meanwhile, the acute aqueous toxicity of reaction solutions to *Photobacterium Phosphoreum* and *Daphnia magna* was rapidly decreased during ozonation. In addition, no obvious difference in the attenuation of TBBPA was found by ozone oxidation using different water matrices, and the effectiveness in natural waters further demonstrates that ozonation can be adopted as a promising technique to treat TBBPA-contaminated waters.

## Introduction

Tetrabromobisphenol A (TBBPA) is a commercially important brominated flame retardant with the highest production volume on the market [1]. It is primarily used as a reactive intermediate in the production of epoxy and polycarbonate resins. Due to its widespread use, high lipophilicity, and persistence, TBBPA has been detected in various environmental samples including air, water, sewage sludge, sediment, aquatic animals, and human tissues [2]. Suzuki and Hasegawa [3] reported the concentrations of TBBPA ranging from 0.3 to 540 ng/L in landfill leachates from five industrial waste sites in Japan, whereas in an influent and effluent wastewater, its concentrations were 130 and 7.7 ng/L, respectively. In another study, Harrad et al. [4] investigated the environmental levels of TBBPA in water, sediments, and fish from nine English lakes, and reported that its concentrations ranged from 0.14 to 3.2 ng/L (water), 0.33 to 3.8 ng/g dry weight (sediment), and < 0.29 to 1.7 ng/g lipid weight (fish). Previous studies suggested that TBBPA can induce hepatotoxicity, cytotoxicity, immunotoxicity, disruption of thyroid homeostasis, and has potential to disrupt estrogen signaling [1,5–7]. For aqueous species, recent investigations indicated that TBBPA exposure could induce developmental toxicity and decrease reproductive success in *Danio rerio* [8,9], and trigger oxidative stress and disrupt enzyme activities in *Carassius auratus* [10,11]. Therefore, it is of great significance to develop methods to efficiently remove TBBPA from the contaminated environment.

Several techniques have been explored in the treatment of TBBPA. TBBPA is refractory to microbial degradation with half-life time ranging from 65 days in aerobic activated sludge to 430 days in anaerobic soil [12]. While high temperature incineration is a traditional method for TBBPA disposal, this process releases highly toxic polybrominated debenzo-*p*-dioxins and dibenzofurans [13,14]. Reductive dehalogenation effectively removes TBBPA, but the debrominated product bisphenol A exhibits higher estrogenic activity than the parent compound [15,16]. TBBPA can be decomposed by simulated solar irradiation [17]. However, this method is impractical because of the high cost of equipment and difficulty in operations. Ozone is a strong oxidizing agent and has been widely used in water treatment for disinfection, increasing biodegradability and efficient removal of a variety of residual pollutants, such as some emerging pharmaceuticals [18–21]. Furthermore, ozonation is advantageous in treatment of both wastewater and drinking water for several reasons: (1) the effective destructive process with the reduced formation of harmful byproducts compared to chlorine; (2) the rapid removal of odorific compounds and organic contaminants in a shorter contact time compared to UV; and (3) the lack of secondary pollution compared to permanganate [21]. Specifically, ozonation has been considered as a promising technique for TBBPA degradation [22]. It was reported that TBBPA at an initial concentration of 50 mg/L could be almost completely removed in 25 min by ozone at pH 9. However, little is known about the reaction intermediates and products formed during ozonation of TBBPA, which hinders the application of this technique in TBBPA treatment.

Molecular modeling has been used to estimate the inherent chemical reactivity at molecular levels, providing information with regard to the reactive sites and prediction of possible reaction intermediates [23–28]. Frontier molecular orbital (FMO) theory is particularly useful in studying the oxidative degradation behaviors of organic molecules [29–31]. This theory postulates the reactivity of the target compounds based on the profiles of the highest occupied molecular orbital (HOMO) and the lowest unoccupied molecular orbital (LUMO) in reacting species, the molecular orbitals that are directly involved in redox reactions.

The present work is designed to investigate the potential of using ozone to degrade TBBPA in water. We first examined the removal efficiency of TBBPA at different pH values. Based on analysis of reaction intermediates and products, the degradation pathway was tentatively proposed. The role of hydroxyl radical during ozonation was determined by using isopropanol as a radical scavenger, and the ozonation products in the presence and absence of isopropanol were compared. The frontier electron densities were calculated to facilitate the identification of the reaction sites toward ozone and hydroxyl radical in TBBPA molecule and the intermediates. In addition, the toxicity of ozonated solution at various reaction times was evaluated using *Photobacterium Phosphoreum* and *Daphnia magna* assay. This study would enhance the general understanding on the ozone-based oxidative process of TBBPA, which may provide useful information for the potential application of ozonation to treat TBBPA-contaminated waters.

## Materials and Methods

### Ethics statement

No specific permits were required for the described field studies: a) no specific permissions were required for these locations/activities; b) locations are not privately-owned or protected; c) the field studies did not involve endangered or protected species.

### Chemicals and reagents

TBBPA (purity: 98%) and commercial humic acid (HA ≥ 90%) was purchased from Aladdin Reagent (Shanghai, China). The high performance liquid chromatograph (HPLC) grade methanol and isopropanol was obtained from Merck Company (Darmstadt, Germany). Ultrapure water was prepared from a Millipore Milli-Q water purification system. Other chemicals used were of analytical grade or higher.

Since TBBPA is sparsely soluble in water, a stock solution of 5 g/L was prepared by dissolving 0.5 g TBBPA in 100 mL of 0.1 M NaOH solution. Stock solutions of other reagents were obtained by dissolving the chemicals directly in ultrapure water. All solutions were stored at 4°C and used within one month.

### Ozonation experiments

Ozonation of TBBPA was performed at room temperature in 100 mL conical flasks, each containing 50 mL of TBBPA solution that was prepared by diluting the TBBPA stock solution in ultrapure water. The initial concentration of TBBPA in the reaction solution was 100 mg/L, higher than those commonly found in aquatic environments to enable the identification of degradation products without preconcentration. Ozone produced by a DJ-Q2020A water fed electrolysis-type ozone generator (Yichang, China) was continuously bubbled into the reactor at a constant flow rate of 36 mL/min through a glass tube with a sintered end. The inlet ozone concentration in the gas phase was measured as 140.6 mg/L using an iodometric method. The reaction solution was perfectly mixed by a magnetic stirrer at 450 rpm. To determine TBBPA removal efficiency at different pHs, the initial pH of each 50 mL solution was adjusted to a desired value by 0.1 M HCl or 0.1 M NaOH solution without using any buffer. To assess the effect of water quality parameters on TBBPA degradation, a series of 50 mL aqueous sample was prepared initially containing 100 mg/L TBBPA and one of the components (nitrate, bicarbonate, sulfate, chloride, and HA). The concentrations were 0.5 and 5.0 mmol/L for anions; 5.0 and 50.0 mg/L for humic acid. Aliquots of 2.0 mL were taken at defined time intervals and analyzed for residual TBBPA. Immediately after sampling, samples were flushed with nitrogen to remove residual ozone. A control experiment was also conducted to check whether TBBPA was removed from the aqueous phase due to physical phenomenon such as volatilization and adsorption. In the control experiment, oxygen instead of ozone was introduced into the reactor at the same flow rate, and the variation of TBBPA concentration was monitored in the same manner.

### Analytical methods

The concentrations of TBBPA during ozonation were analyzed by an Agilent 1200 HPLC equipped with a diode array detector. The separation was performed on a C_18_ reverse phase column (150 × 4.6 mm, 5 μm particle, Zorbax 300SB-C_18_, Agilent, USA), and the column temperature was maintained at 30°C. The mobile phase consisting of water (20%) and methanol (80%) was eluted at 1.0 mL/min. The injection volume was 20 μL, and the detection wavelength was set at 230 nm.

For the identification of ozonation products, an Agilent 1260 Infinity HPLC system was used to deliver the mobile phases of 0.3% formic acid in water (A) and acetonitrile (B) at a total flow rate of 0.2 mL/min. Gradient elution was performed on a Thermo BDS Hypersil C_18_ column (100 × 2.1 mm, 2.4 μm particle) to facilitate the detection of new signals: B = 10% (0–4 min), 60% (4.5–7.5 min), 80% (8–11 min), 90% (11.5–15 min), 100% (15.5–25 min), and 10% (25.5–35 min). The changing of mobile phase proportion was all completed in 0.5 min. Tandem mass spectroscopic analysis was performed on a triple TOF 5600 mass spectrometry (AB SCIEX, USA) equipped with electrospray ionization (ESI) source in negative ion mode. The Q-TOF MS was externally calibrated by automatic infusion of an APCI negative calibration solution (AB SCIEX) using an automated calibration delivery system to ensure the calibration error below 3 ppm and regulate the measured accuracy of the MS and MS/MS data. To be specific, the calibration solution contains eight calibration ions (144.1030, 264.1453, 277.0983, 352.1977, 403.1122, 440.2501, 616.3550 and 792.4598) for TOF MS calibration. As for the MS/MS calibration, 403.1122 was selected as the precursor ion, including five fragment ions (93.0344, 125.0067, 158.0611, 277.0983 and 403.1122), while the operating parameters were set as: declustering potential, −80 V; collision energy, −30 V. Full scanning was acquired under the following conditions: ionspray voltage, −4500 V; source temperature, 550°C; gas 1, 55 arbitrary units; gas 2, 55 arbitrary units; curtain gas, 35 arbitrary units; declustering potential, −80 V; collision energy, −10 V; scan range (*m/z*), 100–1000 amu. Moreover, the fragmentation pattern of each product was obtained by performing product ion scan (MS^2^, *m/z* 70–1000 amu) for structural elucidation. The source/gas parameters for the MS^2^ scan were set the same as those for the full scanning. The collision energies were varied to optimize the sensitivity.

Quantification of bromide and bromate in the reaction mixtures was performed on an ICS-1000 ion chromatograph (Dionex, USA) equipped with a double piston pump and a Dionex DS6 conductivity detector, using a Dionex IonPac AS11-HC analytical column (250 × 4 mm). The flow rate of the eluent (KOH: 20 mM) was 1.0 mL/min. To evaluate the extent of mineralization during ozonation, total organic carbon (TOC) content was measured using a TOC-5000A analyzer (Shimadzu, Japan).

### Toxicity assay

The toxicities of the samples before and after the ozone treatment at different time intervals were assessed using both marine photobacteria *P*. *phosphoreum* and freshwater flea *D*. *magna*. Sample pH for the reaction solution was readjusted to 8.0 using 0.1 M HCl or 0.1 M NaOH solution before toxicity tests (S1 File).

### Computational method

Molecular orbital calculations were carried out at the single determinant (B3LYP/6-311G**) level with the optimal conformation having a minimum energy obtained at the same level in a Gaussian 09 program. The bulk solvent effect of water was considered using the integral equation formalism polarized continuum model (IEFPCM) within the self-consistent reaction field (SCRF) theory [32]. The frontier electron densities (FEDs) of the highest occupied molecular orbital (HOMO) and the lowest unoccupied molecular orbital (LUMO) were determined from Gaussian output files. Values of 2FED^2^_HOMO_ and (FED^2^_HOMO_+FED^2^_LUMO_) were calculated to predict the possible reaction sites for electron extraction and hydroxyl addition, respectively [29].

### Oxidation in real waters

A source water (dissolved organic carbon (DOC) = 9.10 mg C/L, pH 7.49) was taken from Yangtze River in Nanjing section. The raw wastewater (DOC = 45.31 mg C/L, pH 7.74) and secondary wastewater effluent (DOC = 17.04 mg C/L, pH 7.65) was obtained from Nanjing Baguazhou wastewater treatment plant. After vacuum-filtered through 0.22 μm glass fiber membranes, these three waters were stored at 4°C and used within 2 weeks.

Working solutions (100 mg/L) were obtained by diluting the stock with real waters, and the pH was adjusted to 8.0 with HCl solution. The oxidation dynamics of TBBPA by ozone in these real waters were examined following the same procedure as that in ultrapure water. Further, a much lower initial TBBPA concentration (100 μg/L) was prepared to better mimic environmental levels, while still being high enough to permit accurate measurement by the TOF 5600 mass spectrometry without pre-concentration.

## Results and Discussion

### TBBPA removal at various process conditions

**Fig 1:**
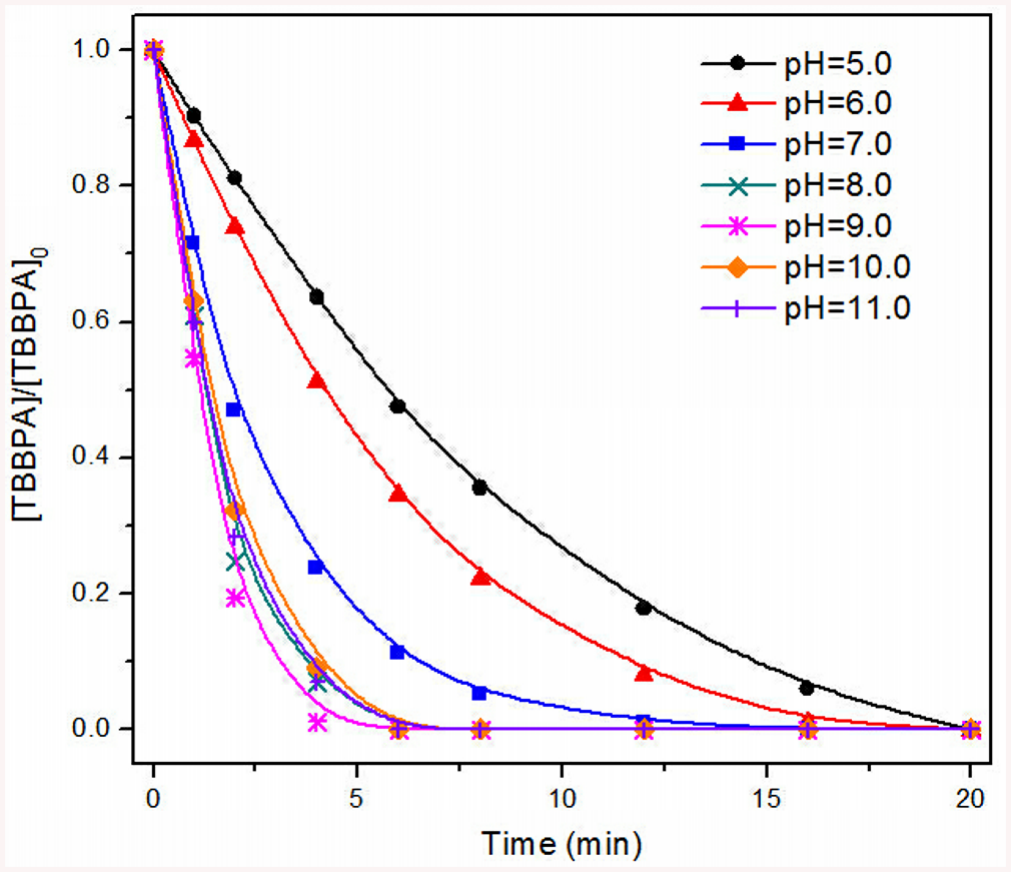
Degradation kinetics of TBBPA at various pH values.

Bubbling of only oxygen was unable to remove TBBPA, excluding any physical process as being responsible for the TBBPA removal during the ozonation process. The degradation of TBBPA at various pH values is plotted as a function of reaction time (Fig 1). Under acidic conditions (pH 5.0 and 6.0), the removal efficiency was relatively low. TBBPA was completely removed after 20 min of ozonation. With the increase of pH, the time for complete removal decreased to 16 min at pH 7.0. The highest removal efficiency was observed at alkaline conditions (pH 8.0, 9.0, 10.0, and 11.0) with complete degradation achieved in about 6 min. The increase of reaction rate at higher pH may result from the deprotonation and dissociation of TBBPA, but can also be related to the enhanced generation of free radicals during ozonation. TBBPA is a hydrophobic compound, and its solubility in aqueous solutions depends on pH. At pH above its p*K*_a_ of 7.4, TBBPA is highly soluble, which reduces the mass transfer resistance, therefore accelerating the reactions with ozone. In addition, it is well known that the reactions between ozone and organics occur in two ways: direct oxidation by ozone and indirect oxidation by hydroxyl radicals. At alkaline conditions, hydroxyl ions will catalyze the decomposition of ozone to •OH radicals. Since •OH has a significantly higher reactivity toward most organic compounds than ozone [33], TBBPA was degraded faster. Considering the solubility of TBBPA and the pH of the natural waters, all further experiments were conducted at pH 8.0.

Since natural waters are complex matrices, possible effects of water components such as inorganic ions and organic matter should not be ignored when ozone is applied. Our results show that the presence of typical anions (SO_4_^2−^, Cl^−^, NO_3_^−^, HCO_3_^−^) and HA did not significantly affect the degradation of TBBPA, while the reason may be due to the excessive O_3_ and/or OH· employed in our tests, which decreased the influence of these substances. Similar finding was also reported in ozonation of bisphenol A by Tay et al. [34].

### Identification of ozonation intermediates and products

Advanced oxidation processes often generate various reaction intermediates and products due to the non-selective nature of hydroxyl radicals. In this study, the intermediates and products formed during the ozonation of TBBPA were identified by the LC-TOF-MS. The high resolution of the TOF-MS allows for accurate mass measurements to determine possible elemental compositions of the molecular ions and their fragments. Structural elucidation of brominated oxidation products was achieved on the basis of their parent ion masses, product ion spectra, bromine isotope patterns, chromatographic retention times, as well as previously proposed degradation intermediates in various oxidative processes for TBBPA [35,36]. After careful examination of the peaks in the chromatograms, a total of 17 intermediates, including 12 organic compounds and 5 inorganic substances, were determined. Detailed information on each product is listed in S1 Table, and the respective MS^2^ spectra are presented in S1 Fig.

### Evolution of organic intermediates and inorganic ions

**Fig 2:**
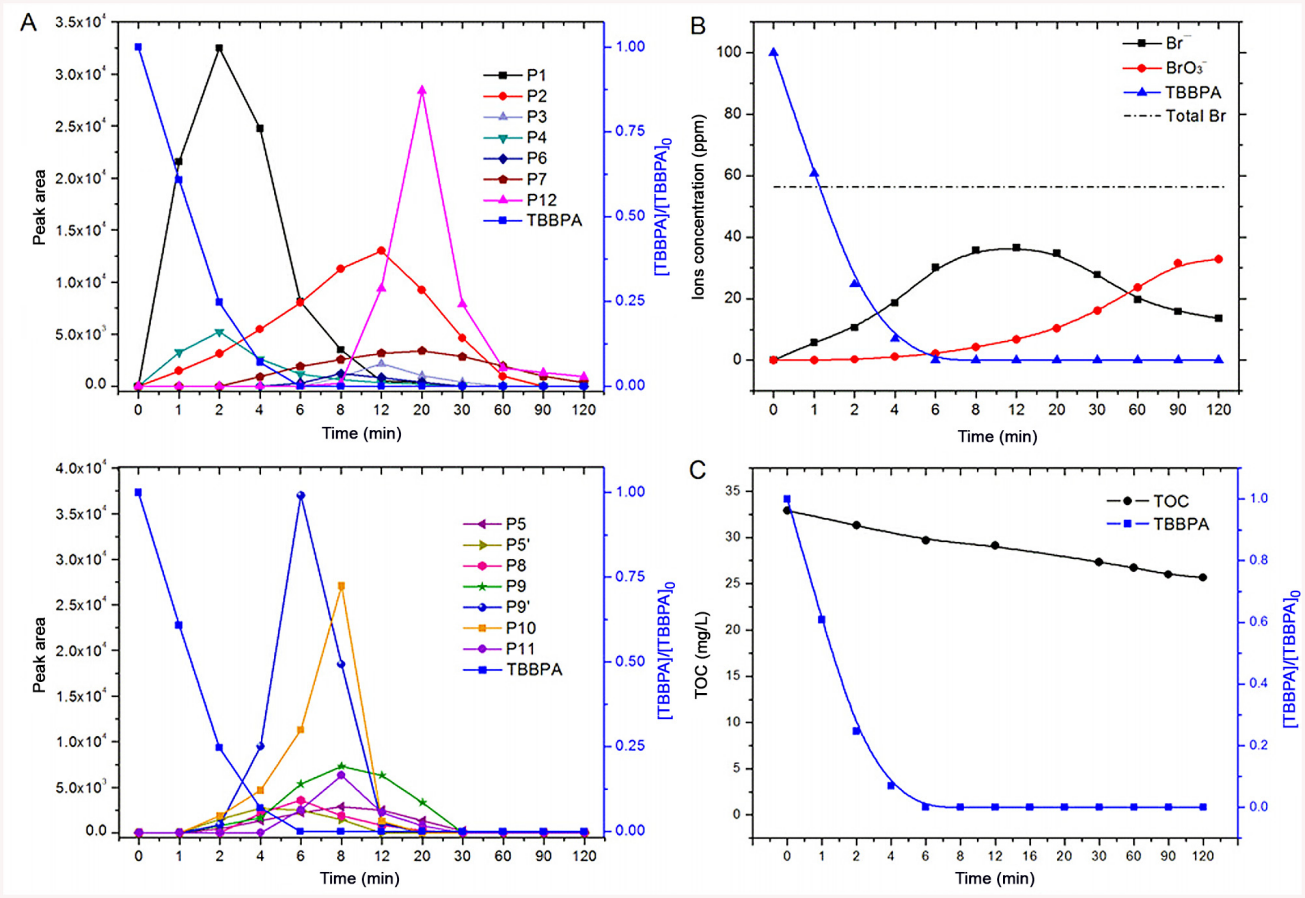
Temporal changes of (A) organic intermediates, (B) inorganic ions and (C) TOC during ozone oxidation of TBBPA. Peak area was recorded by extracting ions in the total ion current chromatogram.

Based on the chromatographic peak area, the evolution profile of the intermediates and products was established in order to get a comprehensive understanding of the reaction processes. Three types of patterns can be discerned in the profiles of intermediate evolution shown in Fig 2A. Clearly, P1 and P4 have a similar pattern (Pattern I). They were detected immediately after the reaction and the yields quickly reached the maxima in 2 min, possibly indicating that they were initially formed intermediates in the transformation of TBBPA. Then, a similar evolution pattern (Pattern II) was observed for another seven compounds (P5, P5', P8, P9, P9', P10, and P11) whose concentrations reached highest in 4–8 min of ozonation. This suggests that the generation of these chemicals in the oxidation process was more difficult than the Pattern I products, probably because Pattern II products were formed via different mechanisms which were kinetically slower such as ring cleavage, or they were not generated directly from the parent compound. Also, it was found that two other oxidation products (P7 and P12) showed a common pattern (Pattern III). These two products did not form initially, and eventually reached to maximum concentration in 20 min. Thus, Profile III products were probably generated from the transformation of Pattern I and II intermediates. Different from all above-mentioned products, product P2 was detected as early as 1 min, and gradually accumulated to the maximum in 12 min followed by a slow disappearance, indicating the easy generation and recalcitrance to further transformation of this product. During the whole reaction process, P3 and P6 always had relatively low mass responses, suggesting that they are minor degradation products of TBBPA. It is noted that the reaction intermediates generally decreased to undetectable levels in 2 h, demonstrating that ozonation is a promising technique for TBBPA removal as well as intermediates elimination.

Fig 2B shows the temporal evolution of inorganic bromine species. It is evident that bromine atoms in aromatic rings were mostly released as bromide (Br^−^) which was subsequently oxidized to bromate (BrO_3_^−^) by ozone. Horikoshi et al. [37] have suggested the formation of bromide ions from TBBPA degradation in UV irradiated alkaline aqueous TiO_2_ dispersions. Since the reductive debromination is not likely to occur in the reaction system of this study, the release of Br^−^ is presumed to be resulted from the benzene ring cleavage. The concentration of Br^−^ reached its maximum (36.6 ppm) in 12 min, and then gradually decreased to 13.5 ppm in 2 h. Compared with Br^−^, the yield of BrO_3_^−^ increased monotonically during the reaction. The concentration pattern in Fig 2B illustrates the sequential formation of Br^−^ and BrO_3_^−^. Consistent with this result, von Gunten [38] also reported the bromate formation from bromide during ozonation of drinking water. In addition, previous studies also indicated the by-product formation during the treatments of bromide-containing water by other methods, such as chlorination [39], UV/persulfate [40] and peroxymonosulfate [41]. These brominated disinfection byproducts mainly include certain bromo-organic products such as bromoform, bromopicrin, dibromoacetontrile and bromoacetone as well as BrO_3_^−^. Notably, BrO_3_^−^ is a category I group B2 carcinogen and is currently regulated at a maximum contaminant level of 10 μg/L in many drinking water regulations by United States Environmental Protection Agency, European Commission standards and World Health Organization guidelines [42,43]. Thus, subsequent treatment methods such as adsorption by ion exchange resin [44] should be implemented to remove this oxyaninon when ozonation is adopted to treat TBBPA-containing waters.

### Mineralization

It was observed from Fig 2C that TOC reduction proceeded much more slowly compared to the degradation of TBBPA. When TBBPA was completely decomposed in 6 min, only 9.8% of TOC was removed. At the end of the 2 h ozonation, the mineralization rate was only 22.0%, with TOC decreasing from 32.91 mg/L to 25.68 mg/L. The relatively low TOC reduction was most likely due to the formation of recalcitrant by-products towards ozonation [21].

### Elucidation of ozonation pathways

**Fig 3:**
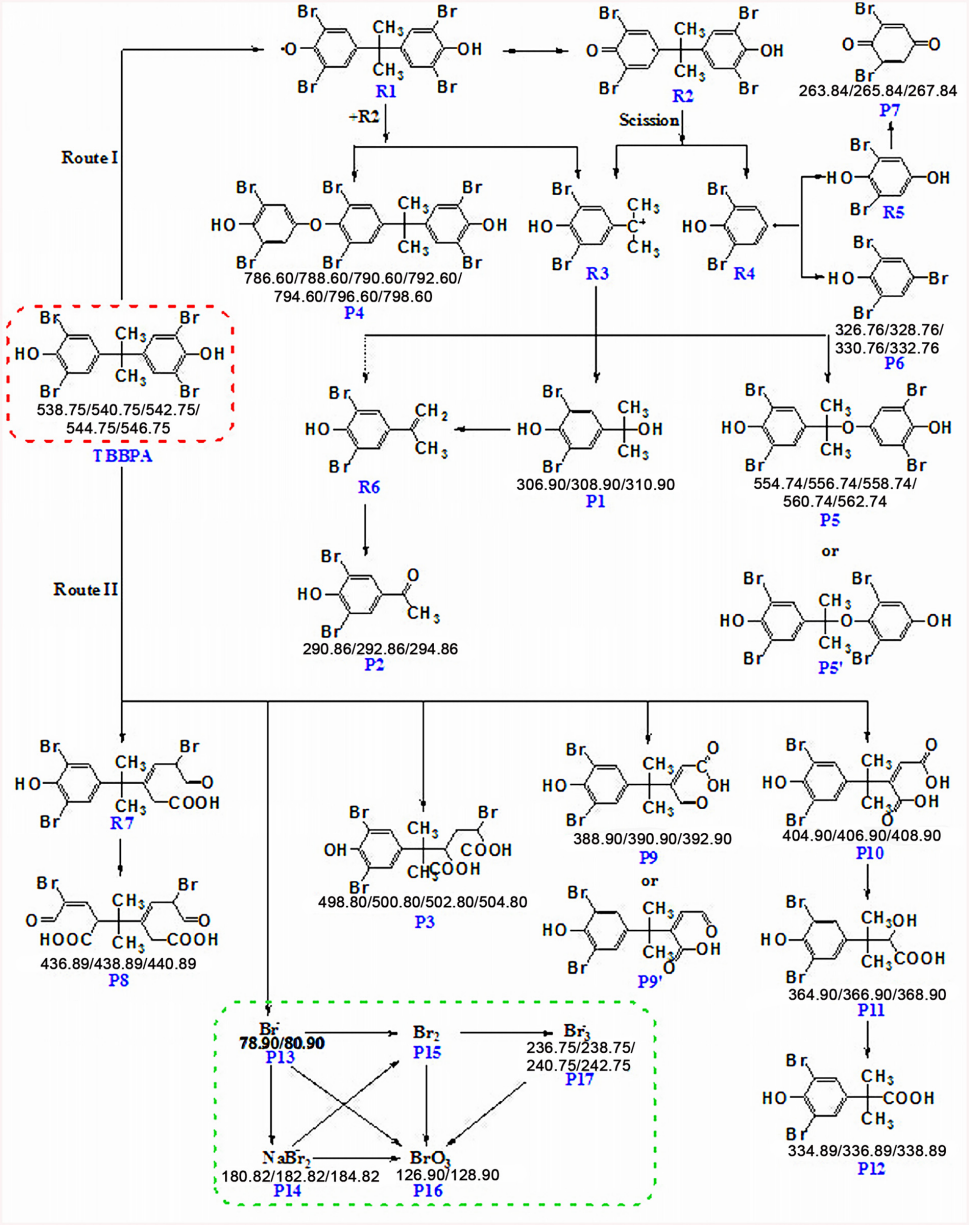
Proposed reaction pathways of TBBPA ozonation. Molecular ion clusters observed in MS spectra for the identified products are given below their respective structures. Possible transformation of inorganic bromine species was shown in green dash line box.

On the basis of the product identification information elaborated above in conjunction with earlier findings on ozone-based oxidation of aromatic compounds [19,21] as well as TBBPA degradation in other reaction systems [35,36,45], the ozonation pathways of TBBPA were tentatively proposed. As shown in Fig 3, two distinct pathways might occur simultaneously during the TBBPA ozonation process.

The first pathway (Route I) is initiated by the deprivation of an electron in the hydroxyl group of TBBPA by O_3_/•OH, leading to the formation of the phenoxy radical R1 which is stabilized by relocating the unpaired electron to the aromatic ring to form R2 through resonance. Coupling of the two TBBPA radicals generates a cationic intermediate R3 and a dimeric product P4 (MW = 794) by the tertiary elimination. Meanwhile, the radical R2 may undergo *β*-scission (cleavage between one of the benzene rings and the isopropyl group) to release R3 and a new radical R4. The carbocation intermediate R3 can be transformed to 4-isopropylene-2,6-dibromophenol (R6) and 4-(2-hydroxyisopropyl)-2,6-dibromophenol (P1) through deprotonation and substitution, respectively. This is in accordance with the observation of Zhong et al. [45], who investigated the heterogeneous catalytic UV/Fenton degradation of TBBPA by titanomagnetite and proposed that the primary reaction involved cleavage of the middle carbon atom. Meanwhile, dehydration of P1 may also generate R6, which was subsequently oxidized to 1-(3,5-dibromo-4-hydroxyphenyl)ethanone (P2, MW = 294). The species R6 has been reported previously in the oxidative degradation of TBBPA as an important intermediate [46]. However, it was not detected in the present work, possibly due to the rapid transformation upon contact with ozone. The R4 radical may become brominated to form 2,4,6-tribromophenol (P6) or couple with hydroxyl radicals resulting in the formation of 2,6-dibromohydroquinone intermediate (R5) which undergone further oxidation to produce 2,6-dibromobenzoquinone (P7). To the best of our knowledge, this is the first report on the occurrence of 2,4,6-tribromophenol in the degradation process of TBBPA and its existence was further confirmed by comparison to an external authentic reference standard. Moreover, the carbocation R3 can substitute a proton of the intermediate R5 with the formation of product P5 or P5' which are isomers (MW = 560). This pathway is largely consistent with the proposed reaction mechanism for the oxidation of TBBPA by aqueous permanganate [36].

In addition to the direct attack on the C-C bond between isopropyl and phenyl, O_3_/•OH can also attack the aromatic bond on benzene ring, as shown in Route II in Fig 3, leading to ring cleavage to form R7, P3, P9, P9', and P10, in company with debrominaton to yield a bromide. A series of complex reactions such as cycloaddition, hydroxylation and decarboxylation are involved in the ring cleavage. P9 and P9' are isomers with the same molecular weight (MW = 392) and similar fragmentation pattern but different retention times. Meanwhile, P10 undergoes decarboxylation and oxidation reactions sequentially to yield P11 and P12. Further oxidation of R7 may also lead to both benzene rings broken to generate P8. It is worth noting that the vast majority of the ring-cleavage products detected in this work were not reported in previous studies on the oxidative degradation of TBBPA, which might be caused by the characteristics of ozone oxidation.

Transformation of inorganic components was observed and presented in the green dash line box in Fig 3. The bromide ions (P13) released in the oxidation process can be transformed into the sodium adduct P14 through combination with sodium ions in the reaction solution, which was further oxidized to form molecular bromine (P15). In addition, direct oxidation of bromide leads to the formation of P15. The intermediate P15 was then quickly oxidized into bromate (P16) or combined with bromide to form P17. Bromate (P16) can also be directly generated from P13 or P17 by oxidation. These reactions are thermodynamically favored according to the standard redox potential (*E* ^ө^). The values of O_3_/H_2_O, BrO_3_^−^/Br_2_, BrO_3_^−^/Br^−^, and Br_2_/Br^−^ are 2.07, 1.52, 1.44, and 1.08 V, respectively [47].

In summary, ozonation of TBBPA is assumed to proceed via two parallel routes. Route I mainly involves the cleavage of the middle carbon atom of TBBPA; while Route II starts by the phenyl ring cleavage concomitant with debromination. Route I generates a cationic intermediate R3 and a radical R4, leading to the formation of P1, P2, P5, P5', P4, P6 and P7. Route II generates ring-opening products such as P3, P8, P9, P9' and P10 via cycloaddition, hydroxylation and decarboxylation reactions, and P10 can be further transformed to products P11 and P12.

### TBBPA ozonation in the presence of radical scavenger

In order to determine the role of hydroxyl radicals, TBBPA ozonation was carried out with isopropanol spiked in the reaction solution. Isopropanol selectively scavenges •OH (1.9×10^9^ M^-1^ s^-1^) to form stable intermediates, thus terminating any radical-induced reactions [48]. If •OH dominated the degradation of TBBPA, addition of isopropanol would inhibit the reaction significantly. The effect of isopropanol on TBBPA degradation is also shown in S2 Fig. It was found that isopropanol had negligible influence as the removal of TBBPA was almost unchanged in the presence of isopropanol at concentration as high as 100 mM (approximately 540 times higher than TBBPA in molar concentration). The results suggested that •OH radicals in fact played an insignificant role in TBBPA degradation. Thus, the faster degradation of TBBPA in alkaline medium is mainly due to the reduced mass transfer resistance resulting from the improved solubility at higher pH values.

With regard to the intermediates and products, P5, P5' and P7 was not detected in the presence of isopropanol, suggesting that hydroxyl radicals are necessary for their generation since the precursor compound R5 was formed via coupling of R4 and •OH, as proposed in Route I. The temporal changes of products P1, P2, P3, P4, P6 and P8 were almost the same as in the non-scavenged system. By contrast, the delayed evolution of the related intermediates and products in Route II (P9, P9', P10, P11, and P12) implies that •OH radicals might facilitate the ring-cleavage and/or ensuing transformation processes (S3 Fig).

### Interpretation by frontier electronic density calculations

**Fig 4:**
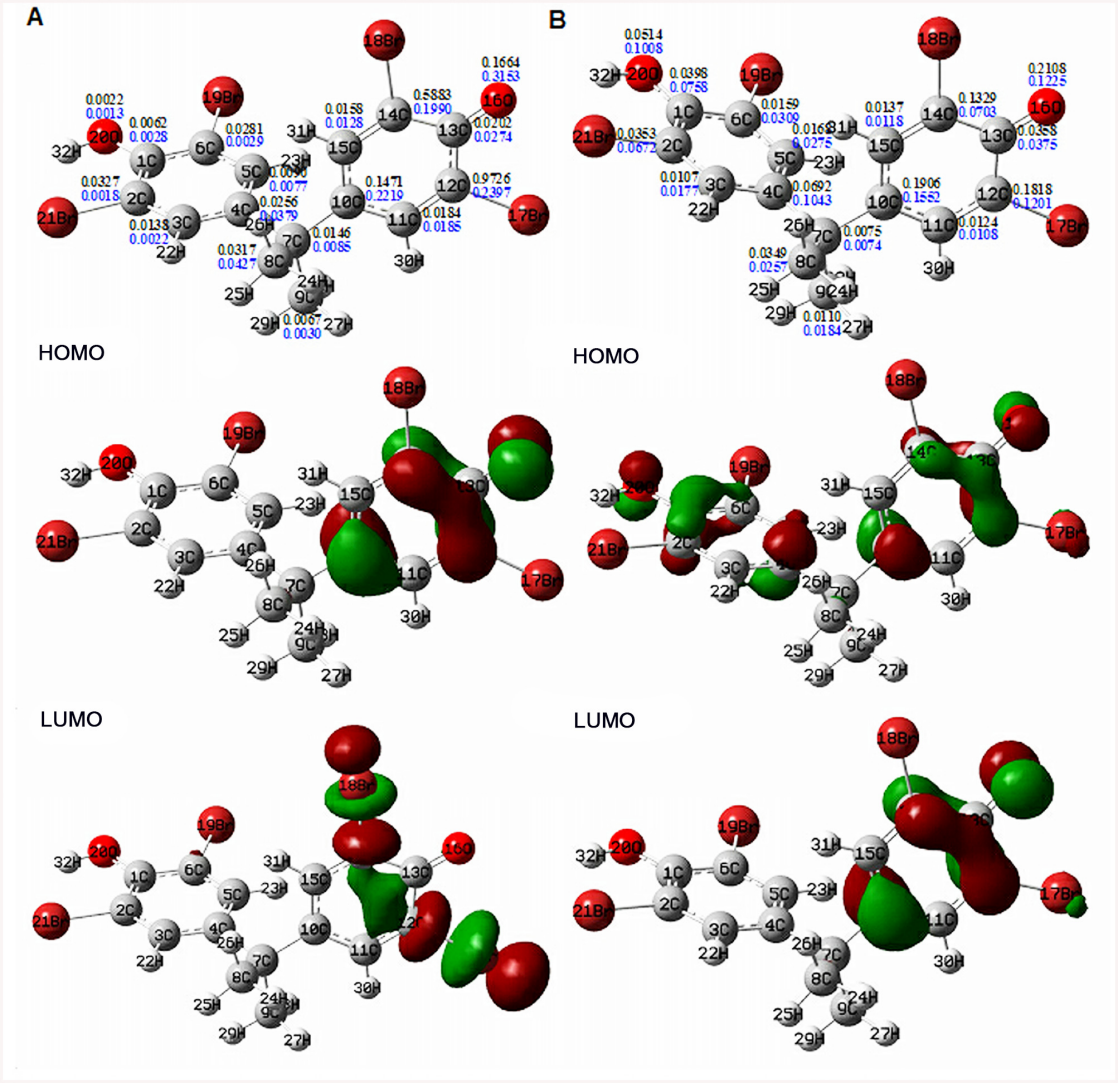
Frontier electron densities on carbon and oxygen atoms of TBBPA anion (A) and TBBPA radical (B). Black number is the FED^2^_HOMO_ + FED^2^_LUMO_ value, and blue number is the 2FED^2^_HOMO_ value. Different colors of ellipsoids in HOMO and LUMO orbitals represent different phases, respectively.

Frontier electronic density (FED) calculations were performed to further rationalize the proposed reaction mechanisms and products. At the studied pH value (pH 8.0), ozone seems to co-exist with hydroxyl radicals in the reaction system [49] and they both contribute to the degradation of TBBPA and the intermediates. To predict the potential reaction sites for electron extraction by ozone or hydroxyl radical, the frontier electron densities of TBBPA were calculated and the results are shown in Fig 4. The three-dimensional iso-surfaces of the HOMO and LUMO frontier orbitals were also displayed to visualize the location of electron density. Here the TBBPA-H form, the predominant species in the reaction solution due to partial ionization of the two acid hydrogens in TBBPA molecule (p*K*_a1_ = 7.4 and p*K*_a2_ = 8.5), was adopted for computations. It can be seen from Fig 4A that O16 has the highest 2FED^2^_HOMO_ value, indicating that O16 should be the first site at which the electron is extracted by ozone or hydroxyl radicals. Thus, the TBBPA anion loses an electron to form the TBBPA radical, which was found to have the highest FED^2^_HOMO_+FED^2^_LUMO_ values at O16 and C10 atoms (Fig 4B). This enables the occurrence of radical attack at the C10 atom by TBBPA radical, hydroxyl radical and bromine atom, yielding the corresponding degradation intermediates P4, R3, R5 and P6. In this regard, the formation of radical R4 appears to be unlikely due to direct radical addition. However, the calculated Wiberg bond orders in the skeleton of TBBPA radical (S4 Fig) shows the C7–C10 bond order was very close to the smallest value located on C7–C4 bond, implying that this bond was also susceptible to bond cleavage with the formation of R3 and R4. In addition to O16 atom, the 2FED^2^_HOMO_ value for C12 and C14 sites in phenyl ring was especially high among all the C atoms, suggesting that these positions were possible attacking points for ozone attack. The products of R7, P9 (P9') and P3 confirmed that O_3_ attacked at C12–C13–C14 semicircle, which can lead to benzene ring opening. Although the C10 has a higher 2FED^2^_HOMO_ value than C14, we think it unlikely that O_3_ will attack this site due to stereo-hindrance effects. Moreover, based on the FED^2^_HOMO_+FED^2^_LUMO_ value, the most reasonable site in TBBPA at which the first addition of a hydroxyl radical could occur was C12. If this predicated reaction occurred, hydroxyl substituted TBBPA (MW = 481) will be the resulting product. However, such an intermediate was not detected in the LC/MS analysis, which may be ascribed to its low concentration. As indicated by the computed FED^2^_HOMO_+FED^2^_LUMO_ values and the visualized molecular orbitals in S5 Fig, further oxidation of these early intermediates all follows the prediction by frontier orbital theory.

### Toxicity assessment

**Fig 5:**
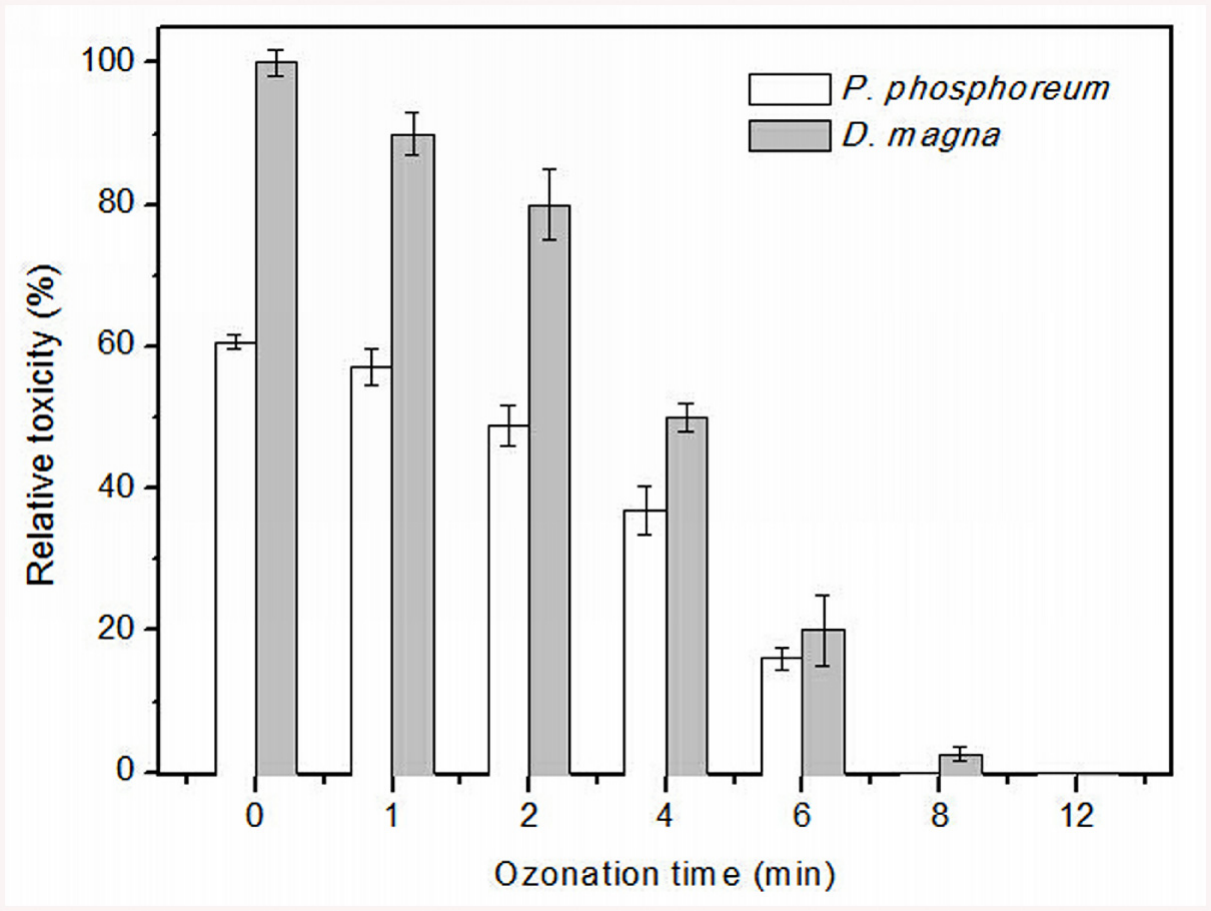
Relative toxicity of reaction solutions to *P*. *phosphoreum* and *D*. *magna* during ozonation of TBBPA. Reaction conditions: [TBBPA]_0_ = 100 mg/L, pH = 8.0.

Since ozonation can possibly generate more toxic intermediates than the parent compound [50,51], it is necessary to measure the change of toxicity during the oxidative process of TBBPA. In this study, toxicity tests were performed using the two frequently used aquatic species *P*. *phosphoreum* and *D*. *magna*. It is illustrated in Fig 5 that the acute toxicity of untreated TBBPA solution was relatively high since the light inhibition was 60.6% for *P*. *phosphoreum*. The toxic effect to *D*. *magna* was particularly remarkable, which reached near 100% immobilization after exposure to the raw solution for 48 h. The change of toxicity upon ozonation was consistent for the two test organisms that the relative toxicity began to decrease from the first minute. When complete TBBPA removal was achieved at 6 min, the relative toxicity decreased to 16.1% and 20% for *P*. *phosphoreum* and *D*. *magna*, respectively. The inhibition rates for *D*. *magna* were always higher than those for *P*. *phosphoreum*, indicating that *D*. *magna* was more sensitive to the reaction mixture. After 12 min, the ozonated samples showed no acute toxicity to the test organisms. The effective elimination of acute toxicity suggests ozonation may be a viable option to treat TBBPA-contaminated waters.

### Oxidation dynamics of TBBPA by ozone in real waters

In addition to being used for the purification of drinking waters, another possible application of ozone is the ozonation of raw wastewater as pretreatment or secondary wastewater effluent as post-treatment to reduce micropollutant input to surface waters. Before ozonation is applied for treating natural waters containing TBBPA, it is advisable to evaluate the effectiveness of this technology. S6 Fig shows the relative residual concentration of TBBPA during treatment of the four different waters as a function of ozonation time. No significant difference in the attenuation of TBBPA was observed for these water matrices, although the DOC value in the raw wastewater reached up to 45.31 mg C/L. Complete TBBPA removal was achieved in 6 min for an initial TBBPA concentration 100 ppm and in as little as 1 min at TBBPA_0_ = 100 ppb. The high similarity in TBBPA decay curves between the ultrapure water and the spiked real waters further confirmed the observations in synthetic water systems that water components generally have little influence on the oxidation rate of TBBPA. However, previous findings showed that the type of water can influence the degradation of phenyl-urea herbicide and pharmaceutical compounds, and the effect is ascribed to the amount of dissolved organic matter (DOM) present in each water [52,53]. Thus, the phenomenon observed in this work may be rationalized by the fact that the ozonation reaction is so fast that DOM or other electron-rich moieties cannot compete with TBBPA for the oxidant.

## Conclusions

The present study suggests that ozonation is highly effective to eliminate TBBPA in aqueous solution. TBBPA at an initial concentration of 100 mg/L was completely decomposed in 6 min of ozonation at pH 8.0. According to the seventeen reaction intermediates and products identified by mass spectrometry analysis and ion exchange chromatography, the reaction pathways mainly involve cleavage of the middle carbon atom and phenyl ring cleavage concomitant with debromination, which was well supported by FED calculations. Radical scavenger experiments revealed that •OH radicals played an insignificant role in TBBPA degradation, while they may influence the generation of some intermediate products. Compared with the elimination of TBBPA, TOC decreased much more slowly, and the mineralization rate was only 22.0% at the end of the 2 h reaction. During ozonation, the reaction mixture showed a rapid decrease in toxicity until became totally harmless to *P*. *phosphoreum* and *D*. *magna* after 12 min. Although the fast removal of TBBPA by ozone oxidation is also demonstrated in real waters, the detection of bromate implies that post-treatment must be launched to remove this toxic oxyaninon in field application of ozonation.

## Supporting Information

S1 Fig Product ion scan spectra of TBBPA and the transformation products. (DOC)

S2 Fig Effect of isopropanol on TBBPA degradation by ozonation. Experimental conditions: [TBBPA]_0_ = 100 mg/L, pH = 8.0.
(DOC)

S3 Fig Evolution of ozonation products at pH 8.0 in the presence of radical scavenger (100 mM isopropanol). The dashed line represents the evolution of the intermediate P9' in the non-scavenged system for comparison.
(DOC)

S4 Fig Wiberg bond order of TBBPA radical calculated by Gaussian 09 program at the B3LYP/6-311G** level. (DOC)

S5 Fig Computed frontier electron densities (FED2HOMO+FED2LUMO) and visualized isodensity surfaces of HOMO and LUMO orbitals for some reaction intermediates. The arrows indicate the possible attacking points. In R3, the nucleophilic reaction usually occurs at positions with highest 2FED^2^_LUMO_ values.
(DOC)

S6 Fig TBBPA transformation in real waters at pH 8.0 as a function of ozonation time for an initial TBBPA concentration of (A) 100 mg/L and (B) 100 μg/L. (DOC)

S1 File The experimental procedures of the acute toxicity tests using *P*. *phosphoreum* and *D*. *magna*. (DOC)

S1 Table Mass measurements obtained by LC-TOF-MS for tetrabromobisphenol A and its identified ozonation products. Compound numbers refer to Fig 3.
(DOC)
