# Supplementary figures and images for: Rapid Removal of Tetrabromobisphenol A by Ozonation in Water: Oxidation Products, Reaction Pathways and Toxicity Assessment
Source: PLoS One. 2015 Oct 2;10(10):e0139580. doi: 10.1371/journal.pone.0139580 (PMC4592209; doi:10.1371/journal.pone.0139580)

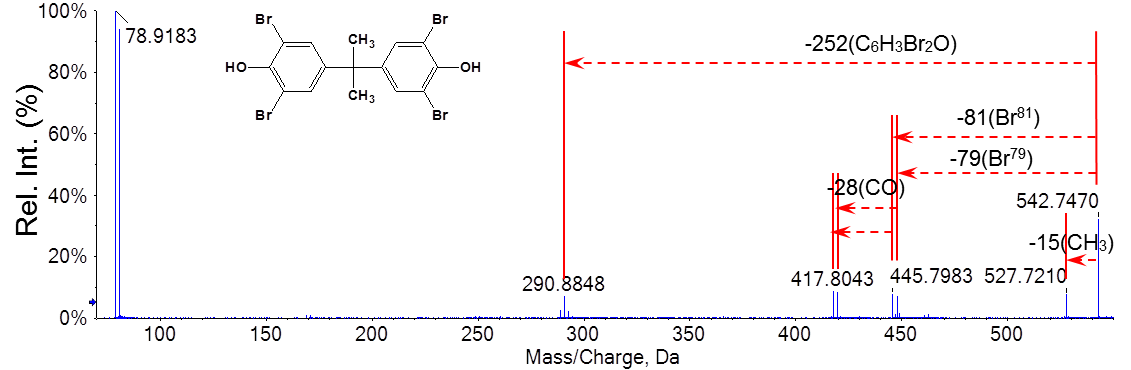


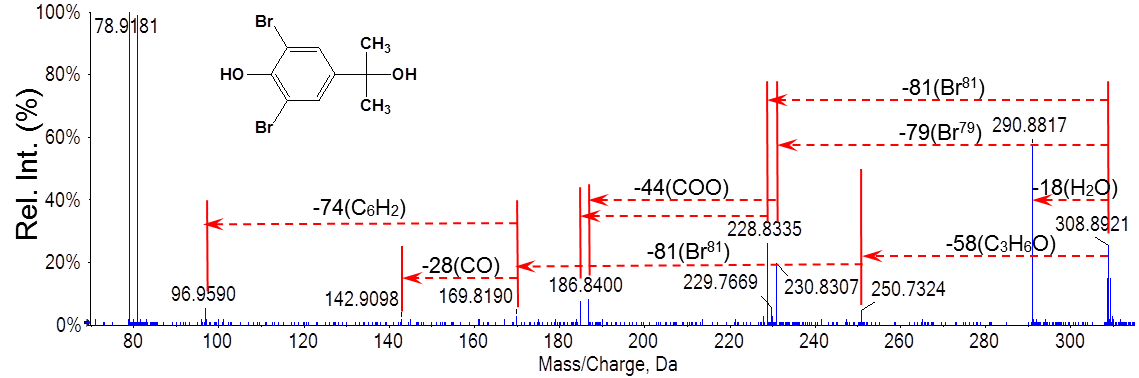


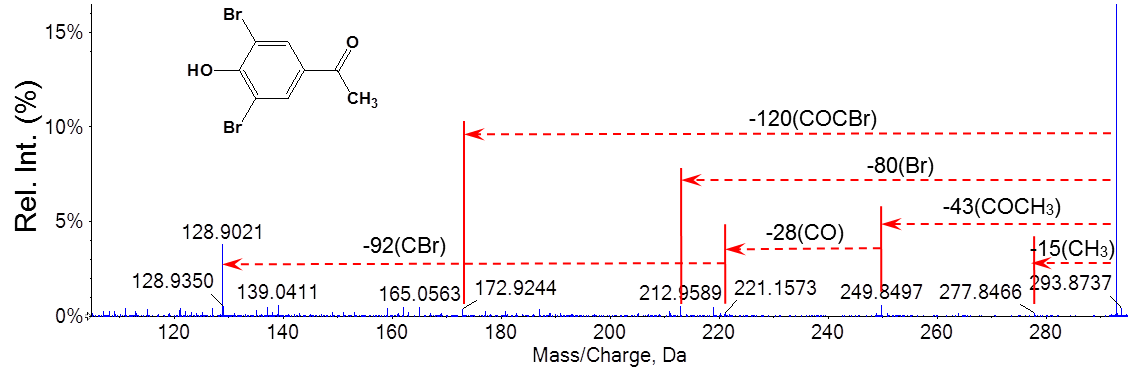


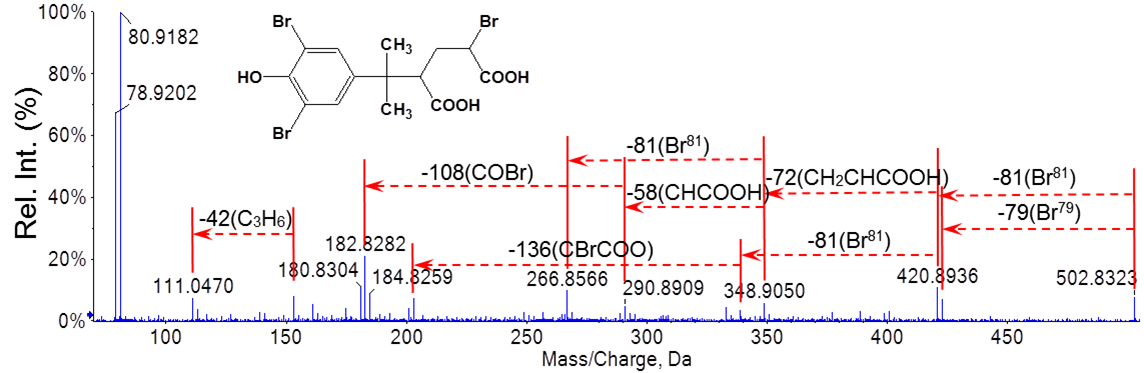


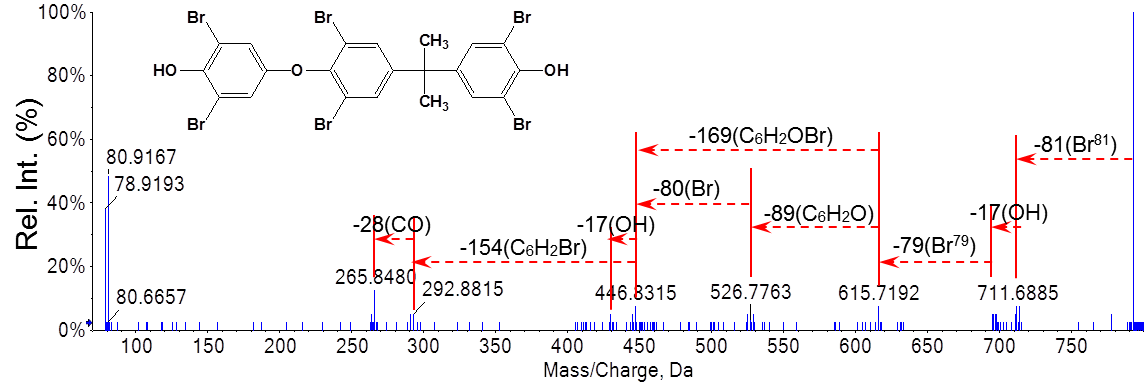


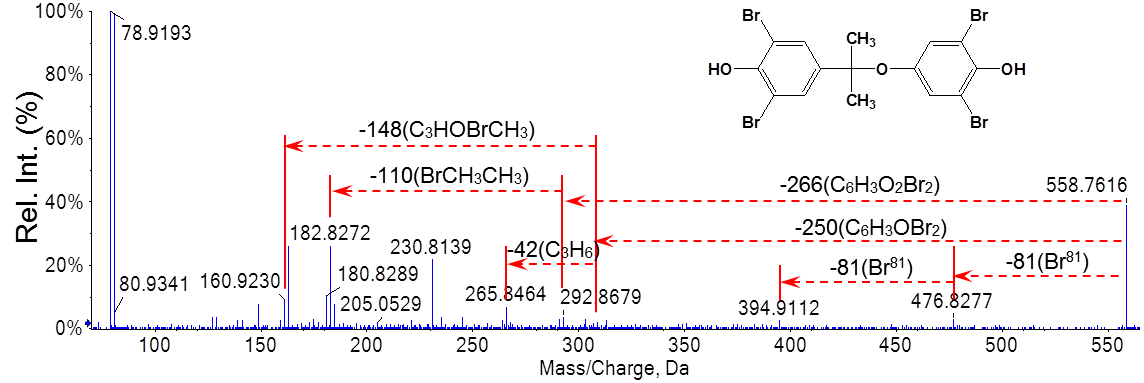


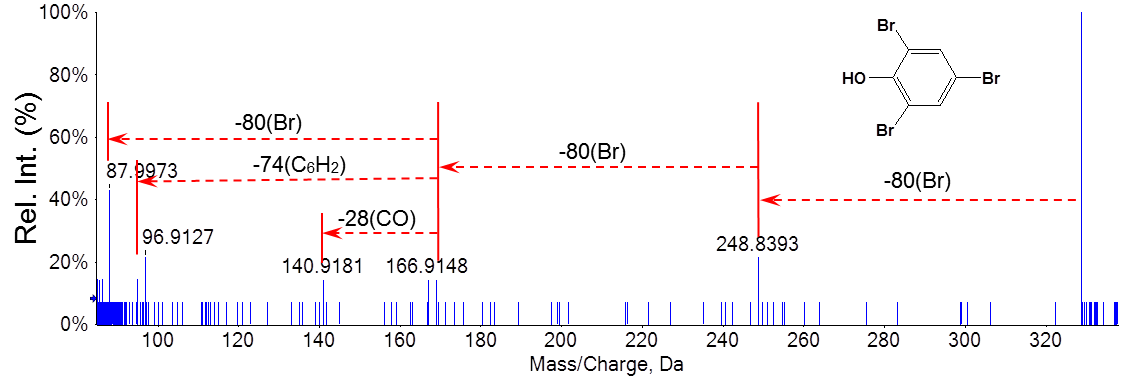


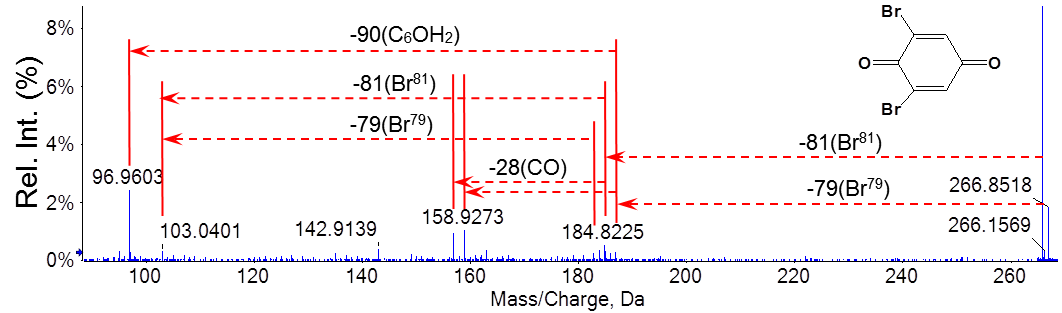


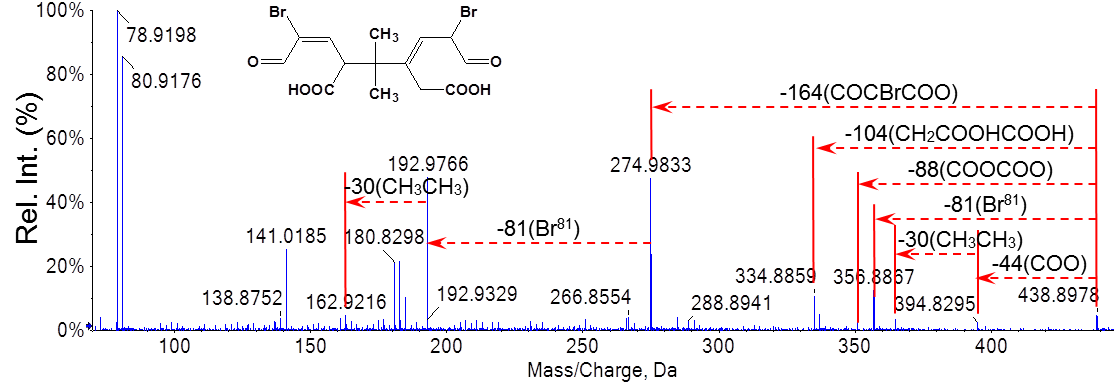


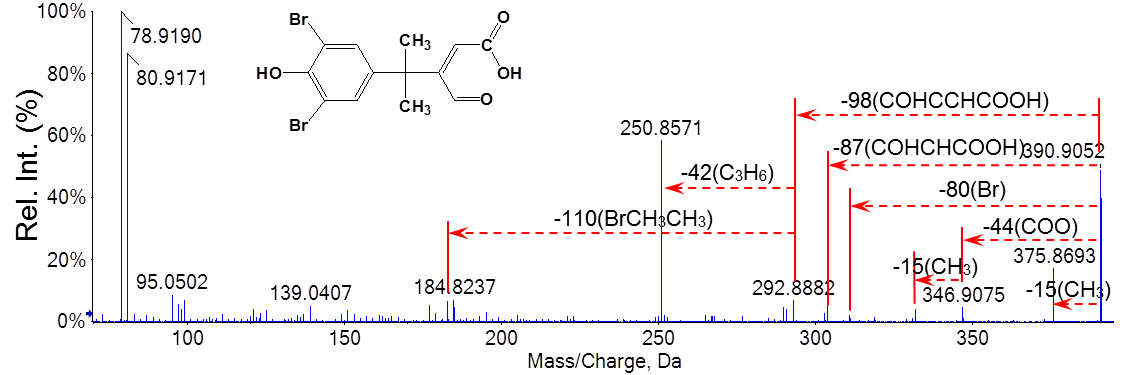


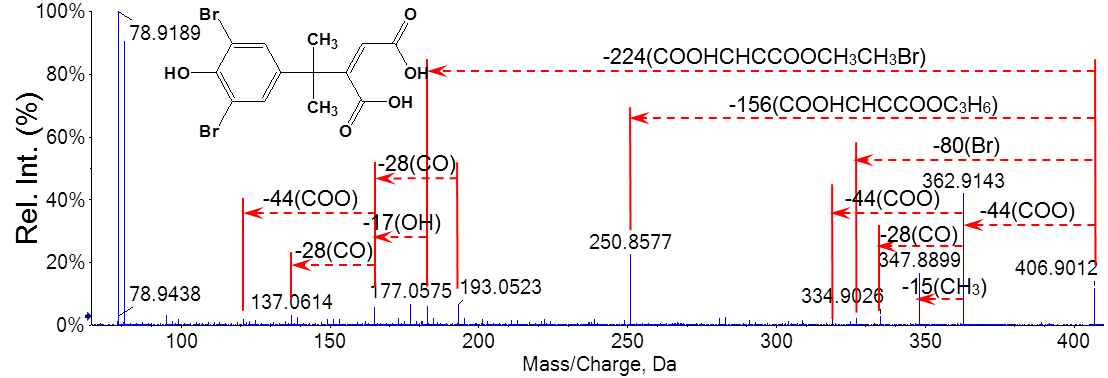


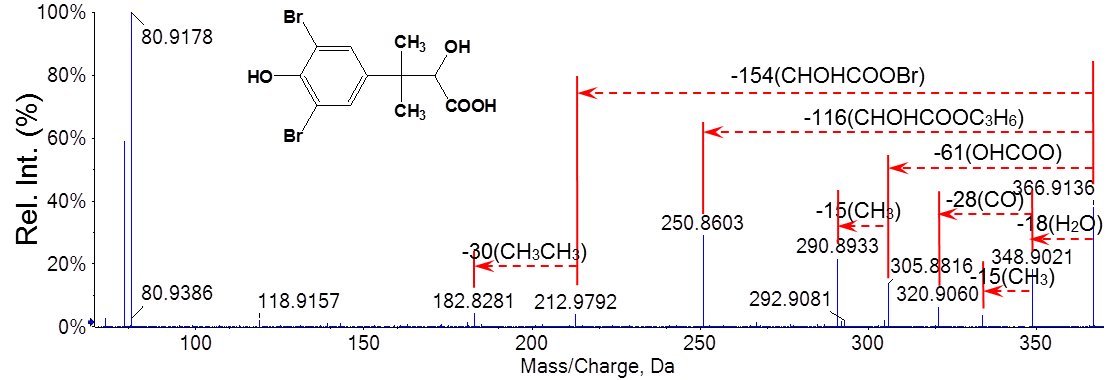


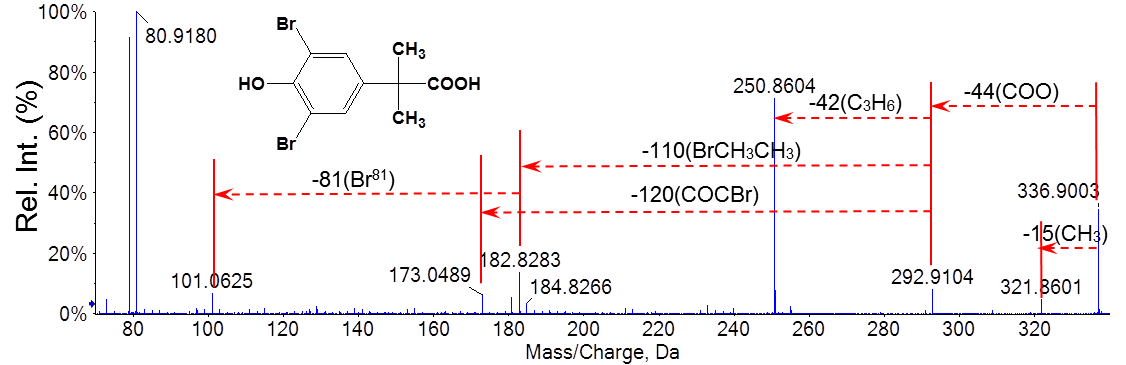


**S1 Fig.** Product ion scan spectra of TBBPA and the transformation products.

Supplement: S1 Fig — (DOC) [file pone.0139580.s001.doc]
