# Supplementary material for: Rapid Removal of Tetrabromobisphenol A by Ozonation in Water: Oxidation Products, Reaction Pathways and Toxicity Assessment
Source: PLoS One. 2015 Oct 2;10(10):e0139580. doi: 10.1371/journal.pone.0139580 (PMC4592209; doi:10.1371/journal.pone.0139580)
Supplement: S2 Fig — Experimental conditions: [TBBPA]0 = 100 mg/L, pH = 8.0. (DOC) [file pone.0139580.s002.doc]

**S2 Fig.** Effect of isopropanol on TBBPA degradation by ozonation. Experimental conditions: [TBBPA]_0_ = 100 mg/L, pH = 8.0.
