# Supplementary material for: Rapid Removal of Tetrabromobisphenol A by Ozonation in Water: Oxidation Products, Reaction Pathways and Toxicity Assessment
Source: PLoS One. 2015 Oct 2;10(10):e0139580. doi: 10.1371/journal.pone.0139580 (PMC4592209; doi:10.1371/journal.pone.0139580)
Supplement: S3 Fig — The dashed line represents the evolution of the intermediate P9' in the non-scavenged system for comparison. (DOC) [file pone.0139580.s003.doc]

**S3 Fig.** Evolution of ozonation products at pH 8.0 in the presence of radical scavenger (100 mM isopropanol). The dashed line represents the evolution of the intermediate P9' in the non-scavenged system for comparison.
