# Supplementary material for: Rapid Removal of Tetrabromobisphenol A by Ozonation in Water: Oxidation Products, Reaction Pathways and Toxicity Assessment
Source: PLoS One. 2015 Oct 2;10(10):e0139580. doi: 10.1371/journal.pone.0139580 (PMC4592209; doi:10.1371/journal.pone.0139580)
Supplement: S4 Fig — (DOC) [file pone.0139580.s004.doc]

**S4 Fig.** Wiberg bond order of TBBPA radical calculated by Gaussian 09 program at the B3LYP/6-311G** level.
