# Supplementary material for: Rapid Removal of Tetrabromobisphenol A by Ozonation in Water: Oxidation Products, Reaction Pathways and Toxicity Assessment
Source: PLoS One. 2015 Oct 2;10(10):e0139580. doi: 10.1371/journal.pone.0139580 (PMC4592209; doi:10.1371/journal.pone.0139580)
Supplement: S5 Fig — The arrows indicate the possible attacking points. In R3, the nucleophilic reaction usually occurs at positions with highest 2FED2 LUMO values. (DOC) [file pone.0139580.s005.doc]

**S5 Fig.** Computed frontier electron densities (FED^2^_HOMO_+FED^2^_LUMO_) and visualized isodensity surfaces of HOMO and LUMO orbitals for some reaction intermediates. The arrows indicate the possible attacking points. In R3, the nucleophilic reaction usually occurs at positions with highest 2FED^2^_LUMO_ values.
