# Supplementary material for: Rapid Removal of Tetrabromobisphenol A by Ozonation in Water: Oxidation Products, Reaction Pathways and Toxicity Assessment
Source: PLoS One. 2015 Oct 2;10(10):e0139580. doi: 10.1371/journal.pone.0139580 (PMC4592209; doi:10.1371/journal.pone.0139580)
Supplement: S6 Fig — (DOC) [file pone.0139580.s006.doc]

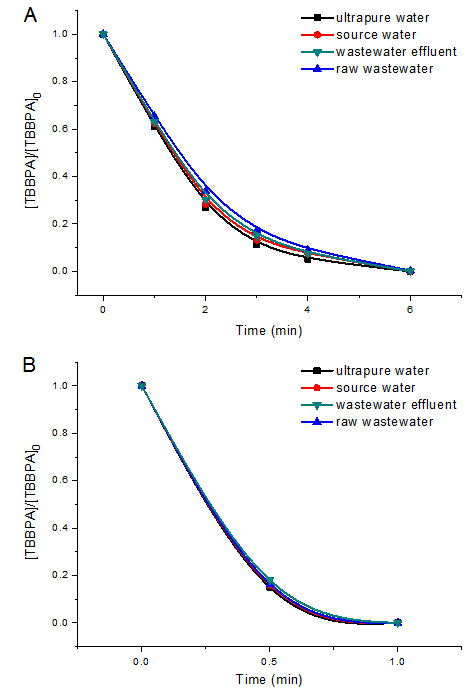


**S6 Fig.** TBBPA transformation in real waters at pH 8.0 as a function of ozonation time for an initial TBBPA concentration of (A) 100 mg/L and (B) 100 μg/L.
