# Supplementary material for: Rapid Removal of Tetrabromobisphenol A by Ozonation in Water: Oxidation Products, Reaction Pathways and Toxicity Assessment
Source: PLoS One. 2015 Oct 2;10(10):e0139580. doi: 10.1371/journal.pone.0139580 (PMC4592209; doi:10.1371/journal.pone.0139580)
Supplement: S1 File — (DOC) [file pone.0139580.s007.doc]

**S1 File.** The experimental procedures of the acute toxicity tests using *P. phosphoreum* and *D. magna*.

The bioluminescence test was carried out according to the standard method of International Standardization Organization (ISO). The sample was applied in its original state without dilution, and the bioluminescence was measured using a Tecan Infinite 200 PRO multimode microplate reader (Tecan, Switzerland) after a 15 min incubation time. Then, relative inhibition in luminescent intensity against the blank control was calculated for each sample. The acute toxicity testing with *D. magna* was conducted following the guideline of Organization for Economic Co-operation and Development (OECD). Ten daphnia neonates (< 24 h) were exposed to 50 mL of sample. Test beakers were maintained at 20 °C with a 16 h: 8 h light: dark photoperiod in an illuminating incubator. No food was provided during the experimental period. After 24 h exposure, immobilization was recorded and expressed as a percentage of the control. To ensure the accuracy, all sample tests and control experiments were performed in triplicate.
