# Supplementary material for: Rapid Removal of Tetrabromobisphenol A by Ozonation in Water: Oxidation Products, Reaction Pathways and Toxicity Assessment
Source: PLoS One. 2015 Oct 2;10(10):e0139580. doi: 10.1371/journal.pone.0139580 (PMC4592209; doi:10.1371/journal.pone.0139580)
Supplement: S1 Table — Compound numbers refer to Fig 3. (DOC) [file pone.0139580.s008.doc]

**S1 Table.** Mass measurements obtained by LC-TOF-MS for tetrabromobisphenol A and its identified ozonation products. Compound numbers refer to Figure 3.

| Compound | RT/min | Molecular formula | Measured mass | Calculated mass | Error/ppm | Proposed structure |
| --- | --- | --- | --- | --- | --- | --- |
| TBBPA | 14.511 | C_15_H_12_Br_4_O_2_ | 542.7468 | 542.7458 | 1.8 |  |
| P1 | 10.712 | C_9_H_10_Br_2_O_2_ | 308.8964 | 308.8955 | 2.9 |  |
| P2 | 11.055 | C_8_H_6_Br_2_O_2_ | 292.8649 | 292.8642 | 2.4 |  |
| P3 | 10.041 | C_14_H_15_Br_3_O_5_ | 500.8043 | 500.8377 | 66.7 |  |
| P4 | 17.663 | C_21_H_14_Br_6_O_3_ | 792.5977 | 792.5911 | 8.3 |  |
| P5 | 10.377 | C_15_H_12_Br_4_O_3_ | 558.7428 | 558.7407 | 3.8 |  |
| P5' | 14.093 | C_15_H_12_Br_4_O_3_ | 558.7428 | 558.7407 | 3.8 |  |
| P6 | 13.449 | C_6_H_3_Br_3_O | 328.7650 | 328.7641 | 2.7 |  |
| P7 | 10.797 | C_6_H_2_Br_2_O_2_ | 265.8417 | 265.8401 | 6.0 |  |
| P8 | 11.93 | C_14_H_16_Br_2_O_6_ | 438.8878 | 438.9221 | 78.1 |  |
| P9 | 10.228 | C_13_H_12_Br_2_O_4_ | 390.9025 | 390.9010 | 3.8 |  |
| P9' | 11.338 | C_13_H_12_Br_2_O_4_ | 390.9015 | 390.9010 | 1.3 |  |
| P10 | 10.551 | C_13_H_12_Br_2_O_5_ | 406.8977 | 406.8959 | 4.4 |  |
| P11 | 10.452 | C_11_H_12_Br_2_O_4_ | 366.9022 | 366.9010 | 3.3 |  |
| P12 | 9.965 | C_10_H_10_Br_2_O_3_ | 336.8906 | 336.8904 | 0.6 |  |
| P13 | 1.076 | Br^−^ | 78.9181 | 78.9189 | 10.1 | Br^−^ |
| P14 | 1.076 | NaBr_2_^−^ | 182.8247 | 182.8249 | 1.1 | NaBr_2_^−^ |
| P16 | 1.076 | BrO_3_^−^ | 126.9042 | 126.9036 | 4.7 | BrO_3_^−^ |
| P17 | 1.076 | Br_3_^−^ | 238.7537 | 238.7535 | 0.8 | Br_3_^−^ |
